# Nanoceria Prevents Glucose-Induced Protein Glycation in Eye Lens Cells

**DOI:** 10.3390/nano11061473

**Published:** 2021-06-01

**Authors:** Belal I. Hanafy, Gareth W. V. Cave, Yvonne Barnett, Barbara K. Pierscionek

**Affiliations:** 1 School of Science and Technology, Nottingham Trent University, Clifton Lane, Nottingham NG11 8NS, UK; gareth.cave@ntu.ac.uk; 2 Faculty of Heath, Education, Medicine and Social Care and Pharmaceutical Research Group, Medical Technology Research Centre, Anglia Ruskin University, Cambridgeshire CB1 1PT, UK; yvonne.barnett@aru.ac.uk; 3 School of Life Science and Education, Staffordshire University College Road, Stoke on Trent ST4 2DE, UK

**Keywords:** cerium oxide nanoparticles, glycation, cataract, glutathione, endocytosis

## Abstract

Cerium oxide nanoparticles (nanoceria) are generally known for their recyclable antioxidative properties making them an appealing biomaterial for protecting against physiological and pathological age-related changes that are caused by reactive oxygen species (ROS). Cataract is one such pathology that has been associated with oxidation and glycation of the lens proteins (crystallins) leading to aggregation and opacification. A novel coated nanoceria formulation has been previously shown to enter the human lens epithelial cells (HLECs) and protect them from oxidative stress induced by hydrogen peroxide (H_2_O_2_). In this work, the mechanism of nanoceria uptake in HLECs is studied and multiple anti-cataractogenic properties are assessed in vitro. Our results show that the nanoceria provide multiple beneficial actions to delay cataract progression by (1) acting as a catalase mimetic in cells with inhibited catalase, (2) improving reduced to oxidised glutathione ratio (GSH/GSSG) in HLECs, and (3) inhibiting the non-enzymatic glucose-induced glycation of the chaperone lens protein α-crystallin. Given the multifactorial nature of cataract progression, the varied actions of nanoceria render them promising candidates for potential non-surgical therapeutic treatment.

## 1. Introduction

Cataract is the loss of the transparency in the eye lens resulting in the scattering and/or absorbance of light and manifesting as blurred vision or glare that can be severely detrimental to sight [1,2,3,4]. Cataracts represent approximately half of the cases of registered blindness worldwide mainly affecting populations over 60 years of age [5,6]. Although many factors have been associated with cataract, it is predominantly an age-related disorder as ageing represents the major risk factor for its development [4]. The most common cataract is referred to as senile cataract. The loss of lens transparency is thought to be caused by the aggregation of lens proteins (crystallins) as the lens ages, and this may be linked to a progressive loss of crystallin solubility [1,4,7,8,9]. Aggregation of lens proteins can be induced by a number of post-translational modifications such as oxidation and glycation [1,4,7,10]. These changes occur very slowly over decades. Additionally, with ageing, the chaperone activity of α-crystallin decreases making the other structural proteins more susceptible to aggregation [9,10]. The largest of the crystallins, α-crystallin, is a molecular chaperone with structural similarity to small heat shock proteins (sHsps), and it inhibits protein aggregation by binding to intermediates that are generated during cellular stress or protein translation [11].

Oxidative stress is believed to be one of the major causes for protein modifications and inactivation in the human lens [12]. In the mitochondria, four-electron reduction of oxygen (O_2_) into water (H_2_O) takes place generating energy that is used to form ATP molecules, a process known as oxidative phosphorylation [2]. When oxygen is partially reduced, various reactive oxygen intermediates, commonly known as reactive oxygen species (ROS), are generated [12]. These include superoxide (O_2_^−^), hydroxyl radicals (•OH), and hydrogen peroxides (H_2_O_2_) [12]. ROS can also be introduced from exogenous sources such as smoking, ionising radiation, diet and air pollutants [2]. ROS can slowly damage the protein structure in the lens, and, because of the lack of protein turnover, the damaged crystallin proteins will accumulate and aggregate over time causing the lens to lose its transparency [1,2,4]. Fortunately, the lens possesses a system of enzymatic and non-enzymatic antioxidants such as catalase, superoxide dismutase (SOD) and glutathione (GSH), which are able to neutralise the excess ROS under normal physiological conditions. However, with age, these natural defence mechanisms can become less efficient, rendering the lens more vulnerable to oxidative damage [13]. There is a growing interest in therapeutics that can maintain this ROS/antioxidant balance with ageing to retain normal lens function and to delay, and ultimately avoid, cataract progression. The need for this is becoming ever more important as advances in medicine and healthcare services have led to an increase in the average lifespan worldwide and hence in more individuals living longer with cataract. With surgery remaining the only effective treatment for cataract, it is necessary to find new therapeutic modalities. It has been predicted that delaying the onset of cataract by 10 years would result in a decreased need for surgery by 50% [6,14].

Cerium oxide nanoparticles, or nanoceria, present an opportunity to act as a cataract therapeutic because of their versatile and recyclable antioxidant properties. The oxidation of cerium creates cerium oxide (CeO_2_) crystals with a face-centred cubic fluorite structure that possesses remarkable redox and catalytic properties [15]. In CeO_2_ crystals, cerium atoms can exist in two different oxidation states, i.e., reduced (Ce^3+^) and oxidised (Ce^4+^) [16]. The stable form is the oxidised state where each cerium atom is coordinated with eight neighbouring oxygen atoms, and each oxygen atom is coordinated with four neighbouring cerium atoms [17]. However, surface defects are normally present on the surface of the crystals and these are associated with Ce^3+^ existence and oxygen vacancies [17]. Recently, advanced X-ray absorption experiments suggested an absence of Ce^3+^ on the surface of nanoceria crystals and that the activity may be more dependent on surface hydroxyl species [18]. The co-existence of Ce^3+^, Ce^4+^ and O_2_ vacancies in the same crystal lattice enables the nanoparticles to act in a versatile way in a redox reaction as either an oxidiser or a reducer depending on the environment. In another words, the electronic configuration of cerium can alternate between Ce^3+^ and Ce^4+^ to best fit the environment [15]. When CeO_2_ loses oxygen in the process of forming surface Ce^3+^, the formed crystal retains its original fluorite structure and is often referred to as CeO_2−*x*_ where *x* is the number of oxygen vacancies [17]. Moreover, the redox properties of cerium oxide nanoparticles have been found to mimic key natural antioxidant enzymes such as superoxide dismutase (SOD) and catalase [19,20].

In our previous work [21], we demonstrated that a novel derivatised ethylene glycol coated nanoceria formulation (EGCNPs) with optimised aqueous colloidal stability, protects human lens epithelial cells (HLECs) against H_2_O_2_-induced oxidative stress when used with a low concentration (50 µg/mL). Additionally, a comprehensive cytotoxicity study was carried out on EGCNPs where it was shown that the formulation was safe up to a concentration of 200 µg/mL on various cellular health parameters including cellular proliferation, basal ROS level, mitochondrial morphology, mitochondrial membrane potential, DNA integrity, and apoptotic and necrotic hallmarks [22]. Mitochondrial uptake of EGCNPs in HLECs was also demonstrated, but the mechanism of uptake was not investigated [22].

In this study, we investigate the mechanism by which EGCNPs can enter HLECs at the established safe concentration and if such entry will act as a nanozyme after actively inhibiting the catalase enzyme in the cells.

## 2. Materials and Methods

### 2.1. Nanoparticles Synthesis and Characterisation

Cerium oxide nanoparticles coated with a hybrid coating (ethylene glycol and ethylene glycol mono- and diacetates, EGCNPs) were synthesised using the aqueous ammonia co-precipitation method starting from cerium(III) acetate hydrate (99.9%, Sigma Aldrich, Gillingham, UK) as reported previously [21]. EGCNPs were characterised by transmission electron microscopy (TEM, JeoL-2010, Tokyo, Japan), powder X-ray diffraction (PXRD, Malvern Panalytical, Malvern, UK), dynamic light scattering (DLS, NanoPlus, Particulate Systems, Atlanta, GA, USA), thermogravimetric analysis (TGA, TGA 4000, PerkinElmer, Waltham, MA, USA), gas chromatography-mass spectroscopy (TGA-GC-MS, GC Clarus 580 – MS Clarus SQ 8 S, PerkinElmer, Waltham, MA, USA), Fourier-transform infrared spectroscopy (FTIR, Cary 630, Agilent, Santa Clara, CA, USA), and UV–VIS spectroscopy (Cary 8454 UV-Vis, Agilent, Santa Clara, CA, USA) [21].

### 2.2. Cell Culture

Human lens epithelial cells (HLECs) (B3, ATCC^®^ CRL11421™, ATCC, Manassas, VA, USA) were grown in Eagle’s minimum essential media (EMEM) (ATCC^®^ 30–2003) supplemented with foetal bovine serum (20%, Scientific Lab Supplies, Nottingham, UK), penicillin (100 units/mL, Sigma Aldrich, Paisley, UK) and streptomycin (0.1 mg/mL, Sigma Aldrich, Paisley, UK). The cells were incubated at 37 °C with 5% CO_2_ in a humidified environment (95% RH). The experiments were carried out on HLECs in the log growth phase.

### 2.3. Time-Dependent Uptake of EGCNPs in HLECs (ICP-MS Studies)

The cellular uptake of EGCNPs over time was measured employing inductively coupled plasma mass spectrometry (ICP-MS, PerkinElmer NexION 1000, Waltham, MA, USA). HLECs were seeded in 6 well plates in complete EMEM (50,000 cells/well) and allowed to establish for 24 h. The medium was then removed and replaced with fresh medium containing EGCNPs (200 µg/mL). Different plates were used to test the following EGCNPs incubation times: 0 min (control), 15 min and 1, 2, and 4 h. After the specific incubation period with EGCNPs, the medium was removed, and the cells were rinsed three times with PBS to remove any surface-bound EGCNPs. The washed cells were harvested using 500 µL trypsin-EDTA (0.025%, Lonza, Slough, UK) and collected in 20 mL Falcon tubes (3 tubes/replicates per each time point). The number of collected cells in each tube was counted using LUNA-II™ Automated Cell Counter (Logos Biosystems, Gyeonggi, Korea). The cells in each tube were then digested by adding concentrated nitric acid (HNO_3_, 2 mL, 70%, Fischer Scientific, Paisley, UK) to each tube and left for 3 days to ensure complete digestion of EGCNPs. After digestion, the solutions were diluted to a known volume (12 mL) using 1% HNO_3_ and the insoluble material was removed by means of filtration. The concentration of cerium ions in each solution was calculated using ICP-MS (PerkinElmer NexION 1000, Waltham, MA, USA). A cerium standard for ICP (TraceCERT^®^, 1000 ppm Ce in nitric acid, Sigma Aldrich, Paisley, UK) was serially diluted in 1% HNO_3_ to make the following concentrations: 1000, 500, 250, 125, 62.5, 31.25 ppb, and a calibration curve was subsequently generated (Figure S1). For accurate determination of cerium content in the cells, the amount of cerium in each condition (tube) was normalised to the counted number of digested cells and the results were expressed as cerium amount (ng) per 10,000 cells.

### 2.4. Mechanism of EGCNPs Uptake in HLECs

To determine if the uptake of EGCNPs in HLECs proceeds mainly by passive uptake mechanism or energy-dependent endocytosis, two ways of endocytosis inhibition were employed. Briefly, HLECs were seeded in 6-well plates as above and treated with chemical endocytosis inhibitors NaN_3_ (10 mM, Sigma Aldrich, Paisley, UK) and 2-deoxy-D-glucose (2-DOG, 50 mM, Sigma Aldrich, Paisley, UK) for 30 min. After the treatment period, EGCNPs (200 µg/mL) were added to the cells and left for 3 h. The cells were then rinsed with PBS, and digestion and ICP-MS analysis were carried out as mentioned above. In a separate set of experiments, endocytosis inhibition was conducted by incubating the cells with EGCNPs (200 µg/mL) for 4 h at 4 °C instead of using chemical endocytosis inhibitors. All experiments were run in triplicate (*n* = 3).

### 2.5. Catalase-Mimetic Activity of EGCNPs in HLECs

To determine if EGCNPs protect HLECs against oxidative stress by exerting catalase-like actions, the catalase inhibitor 3-amino-1,2,4-triazole (3-AT, Sigma Aldrich, Paisley, UK) was employed as reported in the literature with modifications [19,23]. HLECs were seeded in complete EMEM in 96-well plates (5000 cells/well) and allowed to recover for 24 h. The cells were then preincubated with medium containing EGCNPs (50 µg/mL) for 24 h. A negative control (no treatment) and a positive control (3-AT only) were also used. After the treatment period, the medium was aspirated, and the cells were washed once with PBS and incubated with the catalase inhibitor 3-AT (100 mM) for 24 h. After the treatment period, the medium was aspirated, the cells were washed once with PBS and the viability of the cells was determined using the MTT assay as we reported previously [21]. Cell viability was expressed as % of negative control. The experiment was repeated in triplicate (*n* = 3).

### 2.6. Effect of EGCNPs on GSH/GSSG Ratio

The effect of EGCNPs on the ratio between reduced and oxidised glutathione (GSH/GSSG) in HLECs was determined using GSH/GSSG ratio detection assay kit (fluorometric green, ab138881, Abcam, Cambridge, UK) according the supplier’s instructions. HLECs were seeded in 6-well plates in complete EMEM with a seeding density of 50,000 cells/well and left to establish for 48 h. The medium was then removed and replaced with fresh medium containing EGCNPs (50 µg/mL), and the cells were treated for 48 h. A negative control (medium alone) was used for comparison. After the treatment duration, the cells were washed with cold PBS, lysed in radioimmunoprecipitation (RIPA) lysing buffer (200 µL, Thermo Fisher, Inchinnan, UK) and homogenised in the lysing buffer by tilting the plate back and forth. The cell lysates were then collected in 2 mL prelabelled Eppendorf tubes and centrifuged at 22,000× *g* for 15 min at 4 °C to remove insoluble cell material. The clear supernatants were then collected and transferred to clean Eppendorf tubes kept on ice. Deproteinisation of the samples was carried out by adding 40 µL of trichloroacetic acid (TCA, 100% (*w/v*), Sigma Aldrich, Inchinnan, UK) to all the samples and incubating them on ice for 5 min. The samples were then centrifuged at 12,000 g for 5 min at 4 °C, and the supernatants were transferred to clean Eppendorf tubes. The samples were then neutralised by slowly adding NaHCO_3_ (120 µL, 10%, Fisher Scientific, Loughborough, UK) making sure that the pH does not exceed 7 as GSH can be easily oxidised at pH > 7. The samples were centrifuged again as before, and the deproteinised and neutralised lysates were collected in fresh Eppendorf tubes. To make a calibration curve (Figure S2), GSH standards were prepared by serially diluting a stock solution (10 µM) in the supplied assay buffer (Abcam, Cambridge, UK). To measure reduced GSH concentrations, GSH assay mixture (thiol green solution) (50 µL) was added to an equivalent volume of samples and GSH standards in 96-well plates and left to incubate in the dark at room temperature for 1 h. The fluorescence was then read at ex/em = 490/520 nm using a plate reader (TECAN infinite 200 PRO,TECAN, Männedorf, Switzerland ). Similarly, total glutathione concentrations (GSH + GSSG) were measured, but in this case, GSSG was reduced first to GSH using the supplied GSSG probe (Abcam, Cambridge, UK). GSSG concentration was calculated by subtracting GSH concentration from total glutathione concentration, and GSH/GSSG ratio could be determined for all the samples. Each individual sample and standard were assayed in duplicate and their fluorescences were first corrected by subtracting the blank florescence. The experiment was repeated five times (*n* = 5).

### 2.7. Measuring Antiglycation Properties of EGCNPs on BSA and Bovine α-Crystallin

The ability of EGCNPs to prevent protein glycation was determined by measuring the distinctive fluorescence of advanced glycation end products (AGEs) as previously reported [24]. Briefly, two solutions of bovine serum albumin (BSA, 10 mg/mL) and D-glucose (10 mg/mL) were separately made in PBS (pH = 7). Sodium azide was added to each solution (0.02%, Sigma Aldrich, Paisley, UK) as a preservative. Equal volumes of BSA and glucose solution were mixed in 2 mL Eppendorf tubes with or without treatments. The treatments were as follows: EGCNPs (50, 100 and 200 µg/mL) and aminoguanidine as an anti-glycating agent (10 and 20 mM). The samples were incubated for 3 days at 40 °C. After the incubation period, aliquots were taken from all samples (100 µL) and placed in the wells of F-bottom black 96-well plates (Greiner). The fluorescence intensity of AGEs was measured at ex/em: 300 ± 9 nm/400 + 20 nm using a plate reader (TECAN infinite 200 PRO, Männedorf, Switzerland). The same steps were repeated using bovine α-crystallin (1 mg/mL, Sigma Aldrich, Paisley, UK) and glucose (5 mg/mL) to check for protection against glycation in a lens protein. All experiments were run in triplicate (*n* = 3).

### 2.8. Statistical Analysis

Statistical analysis was carried out using GraphPad prism 7 (version 7.05, Graphpad software Inc., USA, 2018). Where relevant, one-way analysis of variance, ANOVA, followed by the appropriate post hoc test (specified in the figure legends) was used to compare groups with statistical significance set at *p* < 0.05. Error bars are presented as means ± SEM unless otherwise specified. Asterisks were used to denote statistical significance with the following notation system: (*) *p* ≤ 0.05, (**) *p* ≤ 0.01, (***) *p* ≤ 0.001 and (****) *p* ≤ 0.0001.

## 3. Results and Discussion

### 3.1. EGCNPs Characterisation

EGCNPs were fully characterised in our previous work [21]. Briefly, the nanoparticles were spherical with TEM core particle size of 4.0 ± 0.8 nm. The crystallinity was confirmed by powder XRD with a calculated crystallite size (Scherrer equation) of 3.5 nm. FTIR and TGA-GC-MS confirmed the presence of the ethylene glycol and ethylene glycol mono- and diacetates as coating materials. EGCNPs were stable in aqueous media with minimum sedimentation for at least 7 days. The zeta potential was +44 mV in water and −9.7 mV in cell culture media; the low zeta potential in culture media was the result of protein coronae formation. Serum proteins are negatively charged at physiological pH, and hence, their accumulation on the surface of the nanoparticles alters the nanoparticle zeta potential and can affect their uptake behaviour [25]. These dynamics, i.e., changes in surface features that affect nanoparticle aggregation state, need to be heeded when determining levels of cellular uptake. EGCNPs showed monodispersity and unimodal distribution of intensity-weighted mean hydrodynamic diameters as measured by dynamic light scattering [21].

### 3.2. Time-Dependent Uptake of EGCNPs in HLECs and Its Mechanism

Studying the interactions between nanomaterials and biological systems is crucial in understanding how nanomaterials affect system function and, in turn, serves as a basis for improving the design of the nanomaterials to achieve the desired purpose. Biological systems are complex in nature making it challenging to predict the behaviour of a certain material in any given system [26]. Nano-systems present extra challenges caused by their multi-faceted properties (e.g., size, charge, and aggregation state) which can dramatically change their physicochemical properties and hence their uptake in and interactions with biosystems [26]. For example, it was reported in various studies that nanoparticles (specifically larger ones) can cause cell membrane rupture and deformation based on how they are taken up [27,28]. Additionally, for a nanomaterial to work as a drug delivery system, a therapeutic or a diagnostic agent, it has to be able to pass through the cell membranes without causing damage. Nanomaterials can erroneously be deemed safe based on toxicological studies that have not correlated them with their cellular uptake. The lack of toxicity may be simply due to the lack of uptake. Therefore, studying the uptake of nanomaterials is an integral part of understanding their activity and toxicity profiles.

**Figure 1:**
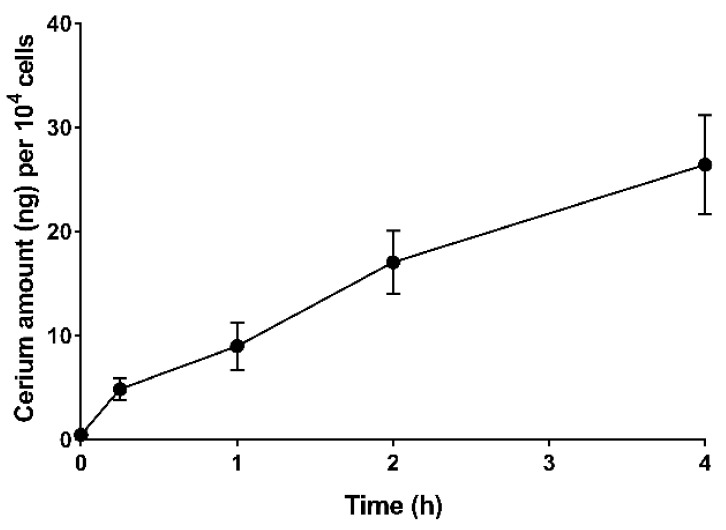
Time-dependent uptake of EGCNPs in HLECs. HLECs were incubated with EGCNPs for 0 and 15 min and 1, 2, and 4 h and the level of internalised cerium in the cells was measured by ICP-MS. The experiments were run in triplicate for each time point and the data are expressed as mean ± standard deviation (SD).

In this work, EGCNPs uptake in HLECs was studied in a time-dependent manner. The amount of cerium oxide internalised by the cells was accurately measured using ICP-MS. Before the analysis, the number of cells in different conditions were counted before preparatory digestion for ICP-MS to ensure highly accurate representation of the results, and to negate any variability that can be caused by slightly different growth pattern in different wells or plates. The highest safe EGCNPs concentration (200 µg/mL), determined from our previous work [21,22] was used in these experiments to ensure the availability of the nanoparticles in the growth medium throughout the specified experiment time. As shown in Figure 1, EGCNPs were rapidly internalised by the cells within only 15 min of incubation (4.8 ng of cerium per 10^4^ cells). The uptake thereafter was shown to be time dependent, increasing to 8.9, 17 and 26.4 ng of cerium per 10^4^ cells after 1, 2 and 4 h, respectively.

**Figure 2:**
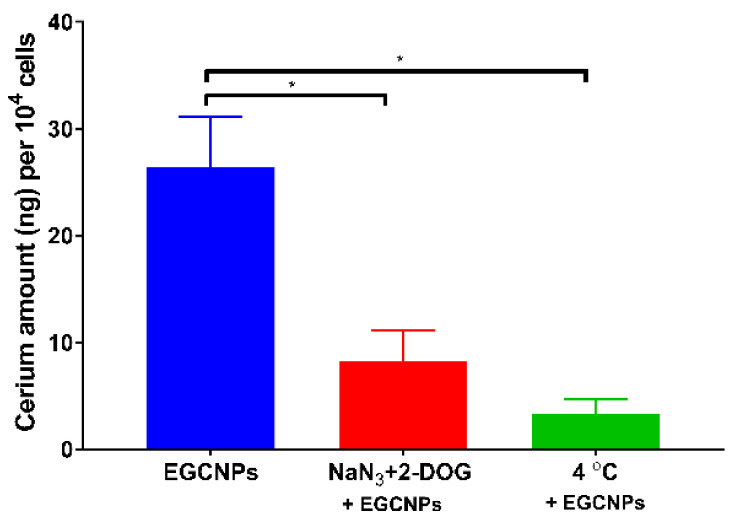
EGCNPs uptake in HLECs for two experimental conditions that inhibit endocytosis compared to negative controls. The reduced amount of cerium in cells for both conditions compared to controls is statistically significant indicating an endocytosis-dependent mechanism of EGCNP uptake. Asterisks denote statistical significance (*n* = 3, *p* < 0.05, one-way ANOVA). Error bars are presented as means ± SD.

To further understand the mechanism of EGCNPs uptake, the cells were incubated with EGCNPs under two different conditions intended to suppress the endocytic pathway. Endocytosis is a main cellular internalisation mechanism for macromolecules, nanoparticles and solutes where a given material is surrounded by a membrane-bound vesicle before being engulfed by the plasma membrane [26]. Contrary to passive cellular pathway, endocytosis is an energy-dependent process for which ATP is needed to proceed [26,29]. Endocytosis can also be subclassified into two key categories: phagocytosis (i.e., cell ingestion for large particles) and pinocytosis (i.e., cell drinking for liquids and solutes) [26,29]. After internalisation, molecules are transported to different cell compartments based on a multitude of factors such as charge, size and interactions with other cellular macromolecules [29]. The first condition was to inhibit endocytosis chemically using sodium azide (NaN_3_) and 2-deoxyglucose (2-DOG) before adding the nanoparticles. These two chemicals deplete the cellular ATP level by blocking the energy-dependent process [30]. The second condition was to incubate the cells at 4 °C, which causes the cell membrane to become more rigid blocking the endocytic process [29]. As shown in Figure 2, significant inhibition of EGCNPs uptake was observed under the two conditions when compared to the negative control.

These results suggest that energy-dependent endocytosis may play a role in efficient uptake of EGCNPs in HLECs. Definitive conclusions about the requirement of energy-dependent endocytosis for uptake of EGCNPs in HLECs would require comparative measurement of ATP amount before and after internalisation. The results may also explain our previously reported data where a slight increase in ATP level in HLECs was observed in the presence of different, safe concentrations, of EGCNPs corroborating the hypothesis that endocytosis was the drive for such an increase [22]. It is noteworthy to emphasise that EGCNPs are also internalised to some extent in the presence of endocytosis-inhibiting conditions (Figure 2), which may suggest that even though endocytosis is the main uptake mechanism, other mechanisms such a passive uptake pathway may be involved. A similar observation was reported for carbon nanotubes where their uptake in multiple cell lines (such as A549, HeLa and Jurkat cell lines) was not predominately dependent on a single pathway [28].

These results present the first reported elucidation of a time-dependent uptake of nanoceria and its mechanism in HLECs. The findings can be compared to results showing uptake behaviour of similar formulations tested in different cell lines. Asati et al. studied the uptake of polymer-coated nanoceria (3–4 nm) carrying different surface charges in multiple cell lines (cardiac myocytes H9c2, human embryonic kidney HEK293, lung cancer A549 and breast cancer MCF-7) [31]. The results showed that positively charged nanoceria were efficiently internalised by all the tested cell lines after 3 h of incubation. However, negatively charged nanoceria were poorly taken up by HEK293, and did not enter H9c2 and MCF-7 cell lines [31]. Confocal studies (which are semi-quantitative compared to ICP-MS) also showed that endocytosis was the main uptake mechanism [31]. Since EGCNPs carry a strong positive charge, it is also likely that this plays a role in its strong uptake by HLECs. Nonetheless, the uptake behaviour of different formulations should be assessed on an individual basis for a given formulation in the cell line of interest given the multitude of the factors that can affect it.

### 3.3. In Vitro Catalase-Like Activity of EGCNPs in HLECs

**Figure 3:**
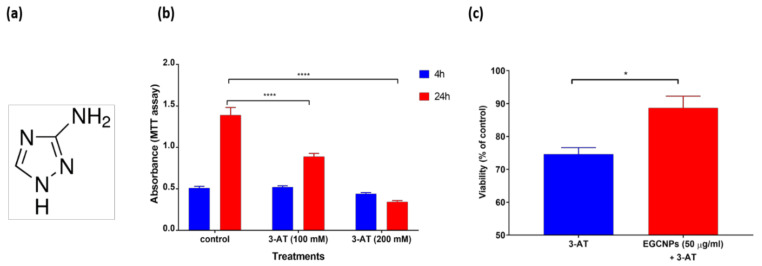
(**a**) Chemical structure of 3-amino-1,2,4-triazole (3-AT), a catalase inhibitor, and (**b**) dose- and time-dependent effect of the catalase inhibitor 3-AT on HLECs viability measured by the MTT assay. Asterisks denote statistical significance from the negative control (two-way ANOVA followed by Tukey’s multiple comparisons test, *n* = 3, **** *p* ≤ 0.0001). (**c**) Catalase-mimetic activity of EGCNPs in HLECs; cells were exposed to 3-AT for 24 h. EGCNPs protect against damage induced by the catalase inhibitor 3-AT (100 mM). The asterisk denotes statistical significance (*n* = 3, *p* < 0.05, Student’s *t*-test). Error bars are presented as means ± SEM.

Catalase is a naturally occurring enzyme in the human lens epithelium [32]. This provides a natural defence mechanism against H_2_O_2_ breaking it down into water and oxygen. When the lens ages, depletion of catalases takes place, which, in turn, increases the propensity of the lens to oxidative insult that can lead to protein post-translational modifications and cataract formation [32]. It was reported that transfecting murine lenses with catalase targeted only to the mitochondria led to significant delay in age-related cataract progression [33]. This provides an opportunity for EGCNPs to act as a catalase mimetic for cataract prevention since they were shown to localise significantly in the mitochondria of HLECs as previously reported in our work [22]. Moreover, 3-AT (Figure 3a) is a well-known catalase blocker causing cellular accumulation of intracellular H_2_O_2_ and cellular death [19]. This anti-catalase activity has been shown to exacerbate various oxidative stress-related conditions such as Alzheimer’s disease, inflammatory diseases and liver dysfunction in both in vitro and in vivo models [20,24,34]. Therefore, in this study, 3-AT was employed to irreversibly block the catalase activity in HLECs and determine the ability of EGCNPs to replace the activity of the inhibited catalase.

First, the effect of two concentrations of 3-AT (100 and 200 mM) on the viability of HLECs was studied at two time points, i.e., 4 and 24 h. As shown in Figure 3b, no decrease in cell viability was observed after 4 h exposure to both concentrations. At 24 h, however, both concentrations (100 and 200 mM) significantly decreased cell viability by 37% and 76%, respectively. Since the lower 3-AT concentration (100 mM) provided a significant viability reduction after 24 h exposure (*p* < 0.0001), it was deemed suitable to use that concentration and exposure duration in studying the catalase-mimetic activity of EGCNPs.

In a subsequent experiment, HLECs were pre-treated with EGCNPs for 24 h before exposure to 3-AT. The results show that the HLECs pre-treated with EGCNPs exerted significant protection against the cytotoxic effect of catalase depletion induced by 3-AT manifested as seen by the increase in viability of 14% for the EGCNP-treated cells (Figure 3c). Such protection, nonetheless, may be partial and requires further investigation to determine its extent. These results suggest that EGCNPs may act as a nanozyme, exhibiting catalase-like activity in HLECs and are worthy of further investigation on the localisation of EGCNPs in the cells to determine whether this could lead to a mitochondrial targeted anti-cataract therapy. This concurs with our previous results in the proliferation studies of HLECs where EGCNPs provided protection against the oxidative insult induced by H_2_O_2_ [21].

### 3.4. Effect of EGCNPs on GSH/GSSG Ratio

**Figure 4:**
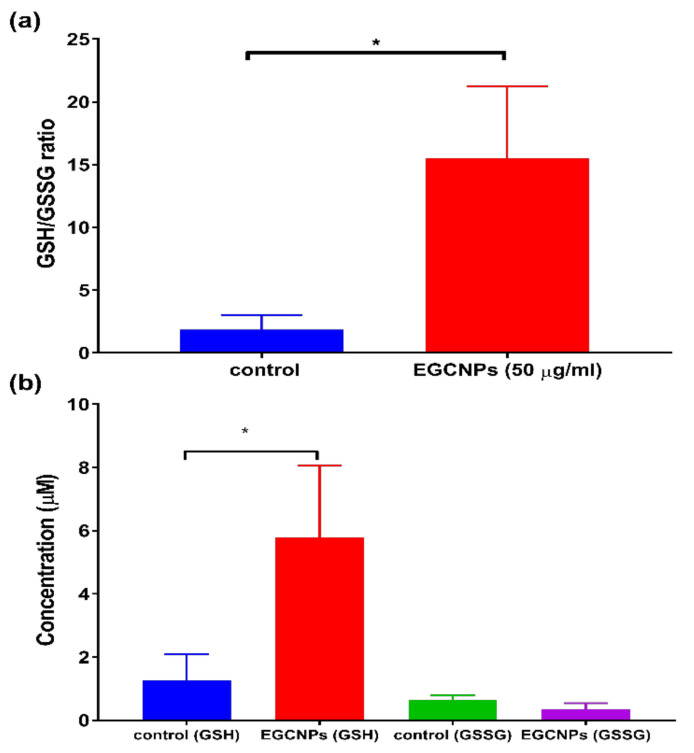
Effect of EGCNPs on GSH/GSSG ratio in HLECs: (**a**) The 48 h exposure to EGCNPs (50 µg/mL) significantly increase GSH/GSSG ratio compared to negative control and (**b**) the effect of EGCNPs (50 µg/mL) on GSH and GSSG concentrations in HLECs after 48 h exposure. The asterisk denotes statistical significance (*n* = 5, *p* < 0.05, Student’s t-test). Error bars are presented as means ± SEM.

Glutathione (GSH) is a tripeptide (*γ*-glutamyl-cysteine-glycine) that plays an important role in the defence system of the human lens against oxidative-stress-induced damage [6,34]. The eye lens naturally contains a high concentration of glutathione in its reduced form (GSH) in addition to a high content of thiol-containing proteins (protein-SH). The cross-linking between such proteins by the formation of disulphide linkages (protein-S-S-protein) is implicated in cataract formation [34]. The presence of reduced GSH in the eye lens protects the thiol content from such cross-linking maintaining the lens transparency by oxidation of the reduced GSH to its oxidised form GSSG [34]. GSH levels in the lens decrease with age with a concomitant increase in disulphide glutathione GSSG [6]. This is reported to be one of the most characteristic features of nuclear cataract formation [2,4]. This loss is also observed in the majority of experimentally induced cataracts [4,34]. Maintaining high levels of GSH in the eye lens can prevent cataract formation. In this study, the effect of EGCNPs on GSH/GSSG ratio in HLECs was investigated after 48 h exposure to the nanoparticles. This was achieved using a sensitive one-step fluorometric assay to quantify reduced GSH content and total glutathione content (GSH + GSSG) from which GSSG content was calculated. The assay employs a non-fluorescent probe that reacts with GSH emitting strong fluorescence that can be quantified using a spectrofluorometer. The total glutathione concentration was measured by enzymatically reducing GSSG to GSH and using the same probe for quantification. The results show that HLECs treated with EGCNPs (50 µg/mL) have a significant increase (*p* < 0.05) in the GSH/GSSG ratio compared to the negative control (Figure 4a).

As shown in Figure 4b, the increase in GSH/GSSG ratio can be primarily attributed to increased content of GSH in EGCNPs-treated cells. Additionally, GSSG concentration in the treated cells was slightly lower but non-significant compared to the control cells. This finding is the first to report the impact of cerium oxide nanoparticles on GSH/GSSG in HLECs. It has been previously reported that cerium chloride-loaded mesoporous silica (CeCl_3_@mSiO_2_) nanoparticles increase GSH content in diabetic rat lenses after 8 weeks of exposure [35]. This may suggest that cerium, as an element, may play a role in promoting the cellular synthesis of GSH. This is worthy of future investigation. Together, these results indicate great potential for EGCNPs in cataract prevention because of not only their direct antioxidant properties but also their GSH promoting effect.

### 3.5. Effect of EGCNPs on Protein Glycation

Protein glycation is one form of protein post-translational modification where sugars and proteins react non-enzymatically leading to the formation of advanced glycation end products (AGEs) [2,35]. The glycation process proceeds very slowly over a period of weeks by a series of reactions between the amino groups of proteins and reducing sugars such as glucose [36]. The reactions start with rapid formation of Schiff base adducts followed by slow Amadori rearrangement and finally formation of AGEs in a complex process known as the Maillard reaction [36]. Crystallins are an ideal target for glycation because of the lack of protein turnover in the lens [1]. The formation of AGEs was shown to be strongly implicated in many pathologies including Alzheimer’s, diabetes, retinopathy, nephropathy, atherosclerosis and cataract formation[1,36,37]. Glycation of the lens proteins has been previously shown to induce protein conformational changes, cross-linking, yellowing and aggregation that are more pronounced with diabetes [1]. In addition to the direct damage to crystallins, sugars can deactivate enzymes including the natural anti-oxidant defence system in the lens (e.g., catalase, superoxide dismutase, glutathione and glutathione reductase), which can lead to the build-up of oxidative stress, a major cause of cataract development [9]. Since oxidative stress is also known to expedite and promote the glycation process, this creates a continuous reaction between AGEs and ROS.

**Figure 5:**
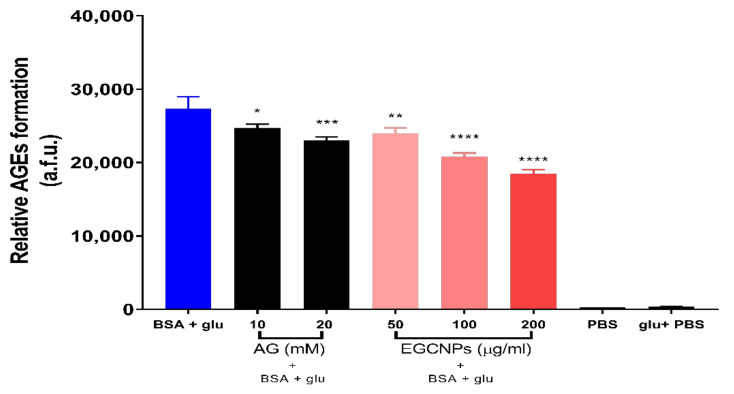
Effect of EGCNPs on the inhibition of the formation of advanced glycation end products (AGEs) for bovine serum albumin (BSA) when incubated with glucose (glu) for 72 h. For comparison, two concentrations of aminoguanidine (AG) were used as it is known for its antiglycation actions. Formation of AGEs was measured by spectrofluorometry using a plate reader at ex/em = 300/400 nm. Asterisks denote statistical significance from BSA + glu, (*n* = 3, * *p* ≤ 0.05, ** *p* ≤ 0.01, *** *p* ≤ 0.001 and **** *p* ≤ 0.0001, one-way ANOVA). Error bars are presented as means ± SEM.

The ability of EGCNPs to prevent protein glycation was tested in vitro by measuring the inhibitory effect of the nanoparticles on the formation of AGEs in a model protein (BSA) when incubated with glucose. BSA was chosen for preliminary testing because of its low cost and the previous glycation data available for BSA [38]. Fluorescence studies were employed to measure formation of AGEs because of the characteristic fluorescence of AGEs [38]. High fluorescence intensity of protein and sugar mixtures is usually indicative of higher degree of protein glycation [38,39]. Three EGCNPs concentrations (50, 100 and 200 µg/mL) were tested, and the antiglycation effect was compared to two concentrations of the standard antiglycation agent aminoguanidine (10 and 20 mM). As shown in Figure 5, all of the tested EGCNPs concentrations showed highly significant inhibition of BSA glycation (**** *p* < 0.0001) when compared to the non-treated protein/glucose mixture.

The anti-glycating effect EGCNPs was concentration dependent with a decrease in formation of AGEs by 13%, 24% and 33% at EGCNPs concentrations of 50, 100 and 200 µg/mL, respectively. The percentage decrease in formation of AGEs for aminoguanidine was 10% and 16% at 10 and 20 mM, respectively. The results clearly demonstrate that EGCNPs can act as a potent anti-glycating agent. It is noteworthy that the glucose concentration employed in this study was significantly higher than the concentration that is naturally found in normal and diabetic lenses (0.7–2.2 and 3–4.5 mM, respectively) [40].

Further investigations were conducted to see if EGCNPs have anti-glycating effects on a commonly abundant crystallin using bovine α-crystallin as the target for testing EGCNPs effects. α-crystallin is not only one of the main structural lens proteins but it also functions as a molecular chaperone that protects other lens proteins and enzymes from aggregation [9,10,11,37].

In addition, α-crystallin is known to be amongst the proteins that are most prone to glycation in presence of sugars [37]. It was shown that α-crystallin protects other proteins such as glutathione reductase and catalase against protein glycation over several days [9]. Such protection was exerted regardless of the species of α-crystallin (bovine or human) [41,42]. Moreover, α-crystallin protects against protein inactivation caused by glucocorticoids, which are a known risk factor for cataract development [9,43], and bovine α-crystallin was reported to fully protect against catalase inactivation induced by 6 day incubation with prednisolone-21-hemisuccinate, a protective effect that was only specific to α-crystallin compared to other control proteins [44].

**Figure 6:**
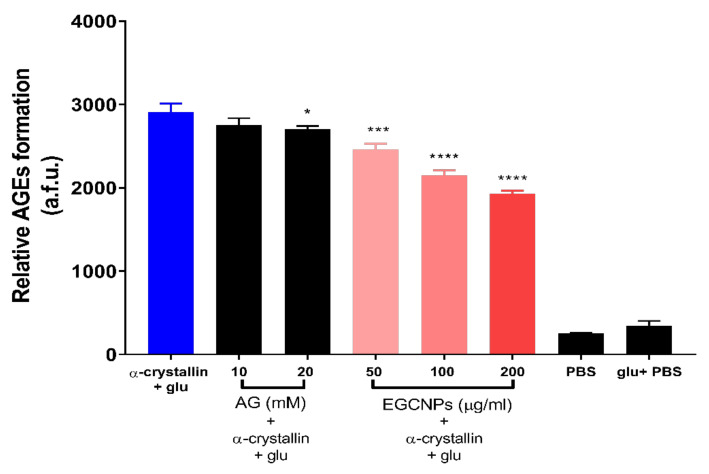
Effect of EGCNPs on the inhibition of the formation of advanced glycation end products (AGEs) for bovine α-crystallin when incubated with glucose (glu) for 72 h. For comparison, two concentrations of aminoguanidine (AG) were used as it is known for its antiglycation actions. Formation of AGEs was measured by spectrofluorometry using a plate reader at ex/em = 300/400 nm. Asterisks denote statistical significance from BSA + glu, (*n* = 3, * *p* ≤ 0.01, *** *p* ≤ 0.001 and **** *p* ≤ 0.0001, one-way ANOVA). Error bars are presented as means ± SEM.

As shown in Figure 6, EGCNPs exhibited significant protective effects against the glycation of α-crystallin after incubation with glucose for 3 days. This is shown to decrease fluorescence of AGEs in all treated conditions compared to non-treated protein and glucose mixtures (Figure 6). Similar to BSA glycation data, EGCNPs protected α-crystallin in a concentration-dependent manner where the mean of AGEs formation was decreased by 15%, 26% and 34% at EGCNPs concentrations of 50, 100 and 200 µg/mL, respectively. Aminoguanidine (AG) also decreased the mean of AGEs formation in α-crystallin by 5% and 7%, respectively, although, unlike the BSA data, the anti-glycating effect of the lower AG concentration was not statistically significant (*p* = 0.09). This could be attributed to the higher glucose/crystallin ratio used compared to glucose/BSA ratio.

Nonetheless, AG (20 mM) showed significant inhibition of AGE formation. Importantly, all tested EGCNPs concentrations exhibited stronger anti-glycation actions compared to the highest AG concentration. It was previously shown that AG slows cataract formation in moderately diabetic rats (plasma glucose < 350 mg/dL), but its actions were overwhelmed in severely diabetic rats (plasma glucose < 350 mg/dL) [45]. It was also suggested that protection against glycation is not enough on its own to resist severe diabetes [2]. The results from different reports in literature indicate that a drug possessing both antioxidant and anti-glycating activities would be more effective than a drug that possess only one of them [2,46]. The data shown here suggest that EGCNPs may present a more preferable alternative given the additional beneficial protective effects of the nanoparticles. Together these results show that EGCNPs have potential in protecting against glucose-induced deactivation of one of the abundant crystallin classes. Preserving α-crystallin activity will, in turn, provide further protection to other structural proteins and antioxidant enzymes in the lens given the chaperone activity of α-crystallin. EGCNPs may protect against the glycation of other lens proteins (β-crystallin and γ-crystallin) because of their structural similarity to α-crystallin [47]. The mechanism by which cerium oxide nanoparticles prevent protein glycation could be ascribed to the activity of surface Ce^3+^ towards many functional groups such as the free amino groups (NH_2_) of α-crystallin that constitute the main targets of glycation [48]. Another possible explanation is that the antioxidant capability of the nanoparticles helps to decrease glycation-promoting free radical in the protein/sugar mixtures. To the best of our knowledge, this is the first report of cerium oxide nanoparticles possessing protective actions against glucose-induced α-crystallin glycation.

## 4. Conclusions

The uptake mechanism and localisation of nanoceria (EGCNPs) have been elucidated for the first time in HLECs. It was demonstrated that safe concentrations of EGCNPs enter HLECs rapidly and in a time-dependent manner. The uptake proceeded mainly by energy-dependent endocytosis. This efficient entry of EGCNPs in HLECs allowed the nanoparticles to exert anticataract actions that were demonstrated in: (1) their ability to replace the activity of the catalase enzyme; (2) improving the ratio between reduced and oxidised glutathione (GSH/GSSG); and finally, (3) their ability to provide a significant protection against the glycation of lens proteins, which is one of the major causes of cataract, particularly in diabetics. Given the multifactorial nature of cataracts, where no single cause is solely responsible for its progression, EGCNPs demonstrated varied actions providing a highly promising alternative for non-surgical intervention.

## Data Availability

Data are available upon the reasonable request from the corresponding author.

## Supplementary Materials

The following are available online at https://www.mdpi.com/article/10.3390/nano11061473/s1, Figure S1: ICP-MS calibration curve (cerium), Figure S2: GSH standards calibration curve (R^2^ is 0.9999) generated using a spectrofluorometer at ex/em = 490/520 nm.
