# Supplementary material for: Nanoceria Prevents Glucose-Induced Protein Glycation in Eye Lens Cells
Source: Nanomaterials (Basel). 2021 Jun 1;11(6):1473. doi: 10.3390/nano11061473 (PMC8228845; doi:10.3390/nano11061473)
Supplement: Supplementary file 1 [file nanomaterials-11-01473-s001.zip › nanomaterials-1229228-supplementary.pdf]

nanomaterials

Supplementary Materials

Nanoceria Prevents Glucose-Induced Protein Glycation in Eye Lens Cells

Belal I. Hanafy [1,*], Gareth W. V. Cave [1], Yvonne Barnett [2] and Barbara K. Pierscionek [2,3,*]

[1] School of Science and Technology, Nottingham Trent University, Clifton Lane, Nottingham NG11 8NS, UK; gareth.cave@ntu.ac.uk
[2] Faculty of Heath, Education, Medicine and Social Care and Pharmaceutical Research Group, Medical Technology Research Centre, Anglia Ruskin University, Cambridgeshire CB1 1PT, UK; yvonne.barnett@aru.ac.uk
[3] School of Life Science and Education, Staffordshire University College Road, Stoke on Trent ST4 2DE, UK
* Correspondence: belal.hanafy2017@my.ntu.ac.uk (B.I.H.); barbara.pierscionek@aru.ac.uk (B.K.P.)

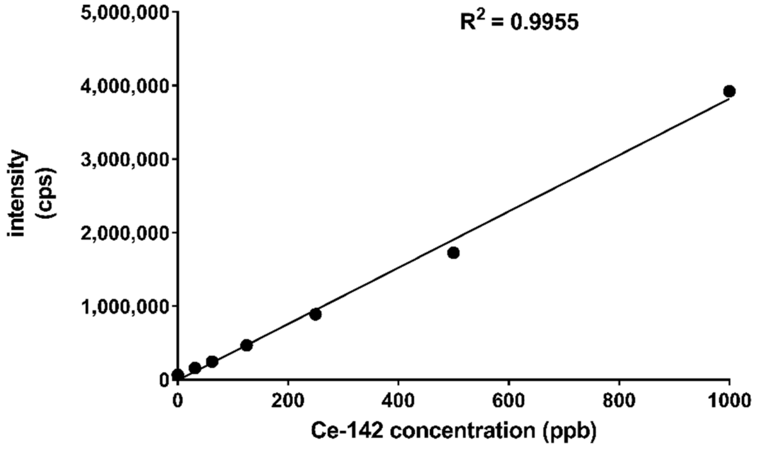

Figure S1. Inductively coupled plasma mass spectrometry (ICP-MS) calibration curve (cerium).

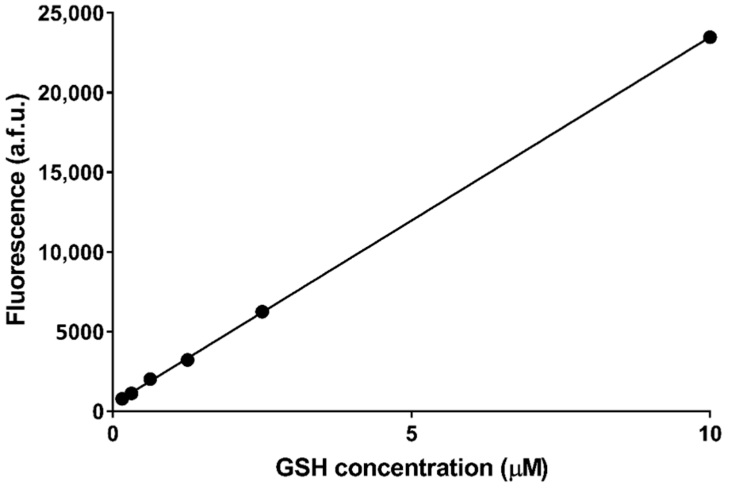

Figure S2. Glutathione (GSH) standards calibration curve (R^2 is 0.9999) generated using a spectro-fluorometer at ex/em = 490/520 nm.
